# Comparative mitogenomics elucidates the population genetic structure of *Amblyomma testudinarium* in Japan and a closely related *Amblyomma* species in Myanmar

**DOI:** 10.1111/eva.13426

**Published:** 2022-06-23

**Authors:** Wessam Mohamed Ahmed Mohamed, Mohamed Abdallah Mohamed Moustafa, May June Thu, Keita Kakisaka, Elisha Chatanga, Shohei Ogata, Naoki Hayashi, Yurie Taya, Yuma Ohari, Doaa Naguib, Yongjin Qiu, Keita Matsuno, Saw Bawm, Lat Lat Htun, Stephen C. Barker, Ken Katakura, Kimihito Ito, Nariaki Nonaka, Ryo Nakao

**Affiliations:** ^1^ Laboratory of Parasitology, Department of Disease Control, Faculty of Veterinary Medicine Hokkaido University Sapporo Japan; ^2^ Division of Bioinformatics, International Institute for Zoonosis Control Hokkaido University Sapporo Japan; ^3^ Department of Animal Medicine, Faculty of Veterinary Medicine South Valley University Qena Egypt; ^4^ Department of Food and Drug Administration Ministry of Health Nay Pyi Taw Myanmar; ^5^ Department of Veterinary Pathobiology, Faculty of Veterinary Medicine Lilongwe University of Agriculture and Natural Resources Lilongwe Malawi; ^6^ Department of Hygiene and Zoonoses, Faculty of Veterinary Medicine Mansoura University Mansoura Egypt; ^7^ Division of International Research Promotion, International Institute for Zoonosis Control Hokkaido University Sapporo Japan; ^8^ Division of Risk Analysis and Management, International Institute for Zoonosis Control Hokkaido University Sapporo Japan; ^9^ International Collaboration Unit, International Institute for Zoonosis Control Hokkaido University Sapporo Japan; ^10^ One Health Research Center Hokkaido University Sapporo Japan; ^11^ Department of International Relations and Information Technology University of Veterinary Science Nay Pyi Taw Myanmar; ^12^ Department of Pharmacology and Parasitology University of Veterinary Science Nay Pyi Taw Myanmar; ^13^ Department of Parasitology, School of Chemistry and Molecular Biosciences The University of Queensland Brisbane Queensland Australia

**Keywords:** *Amblyomma*, cryptic species, mitogenome, phylogeography, population expansion, ticks

## Abstract

Ticks are the second most important vector capable of transmitting diseases affecting the health of both humans and animals. *Amblyomma testudinarium* Koch 1844 (Acari: Ixodidae), is a hard tick species having a wide geographic distribution in Asia. In this study, we analyzed the composition of *A. testudinarium* whole mitogenomes from various geographical regions in Japan and investigated the population structure, demographic patterns, and phylogeographic relationship with other ixodid species. In addition, we characterized a potentially novel tick species closely related to *A. testudinarium* from Myanmar. Phylogeographic inference and evolutionary dynamics based on the 15 mitochondrial coding genes supported that *A. testudinarium* population in Japan is resolved into a star‐like haplogroup and suggested a distinct population structure of *A. testudinarium* from Amami island in Kyushu region. Correlation analysis using Mantel test statistics showed that no significant correlation was observed between the genetic and geographic distances calculated between the *A. testudinarium* population from different localities in Japan. Finally, demographic analyses, including mismatch analysis and Tajima’s *D* test, suggested a possibility of recent population expansion occurred within Japanese haplogroup after a bottleneck event. Although *A. testudinarium* has been considered widespread and common in East and Southeast Asia, the current study suggested that potentially several cryptic *Amblyomma* spp. closely related to *A. testudinarium* are present in Asia.

## 1 INTRODUCTION

Ticks are obligatory ectoparasites that feed on the blood of various vertebrate species including mammals, birds, and reptiles (Brites‐Neto et al., 2015). There are three major tick families described, namely, Ixodidae, Argasidae, and Nuttalliellidae (Horak et al., 2002). Some species of Ixodidae (hard ticks) and Argasidae (soft ticks) are implicated in the transmission of several bacterial, viral, and protozoan pathogens to humans and animals. Ticks are widely distributed around the world, especially in warm humid regions to remain hydrated and subsequently undergo metamorphosis (Vail & Smith, 1998).

*Amblyomma* is the third biggest genus of Ixodidae ticks with more than 130 species (Guglielmone et al., 2014), some of which act as disease vectors of many pathogens, for instance *Rickettsia tamurae* and *Rickettsia raoultii* causing spotted fever (Fournier et al., 2006; Piotrowski & Rymaszewska, 2020), *Ehrlichia chaffeensis* causing ehrlichiosis (Cao et al., 2000), and severe fever with thrombocytopenia syndrome (SFTS) virus causing SFTS in humans (Suh et al., 2016). The geographical distribution of *Amblyomma* species is expanding due to anthropogenic activities, climate change, and increased geographical ranges of animal hosts (Childs & Paddock, 2003). In the United States, *Amblyomma americanum* populations have been constantly enlarging that contributed to change in the epidemiology of spotted fever group rickettsiosis in the country (Dahlgren et al., 2016; Sagurova et al., 2019). In Africa, *Amblyomma* species transmit *Ehrlichia ruminantium* and *Rickettsia africae* that cause heartwater disease and African tick‐bite fever in animals and humans, respectively (Kelly et al., 1996; Yunker, 1996). Furthermore, although very little is known about the diseases caused by pathogens transmitted by this group of ticks (Jabin et al., 2019), there are 14 *Amblyomma* species in Asia (Voltzit, 2002), and at least three species, including *Amblyomma geoemydae*, *Amblyomma helvolum*, and *A. testudinarium* are known to carry pathogens (Fournier et al., 2006; Sumrandee et al., 2014; Takano et al., 2012).

*Amblyomma testudinarium* Koch, 1844 (Acari: Ixodidae), is widely distributed in the Asian countries including Malaysia, India, Thailand, Laos, Vietnam, Indonesia, Borneo, Sarawak, the Philippines, Japan, Korea, Mainland China, and Taiwan (Kang et al., 1981; Lim et al., 2018). In Japan, *A. testudinarium* distribution was limited to the warm areas of Kanto region and the southwestern part of Japan, including Chubu, Kinki, Chugoku, Shikoku, Kyushu, and Okinawa regions (Takada, 1990). In recent years, *A. testudinarium* was infesting on grazing cattle and brown bears in the northern parts of Japan such as Aomori and Hokkaido prefectures, which were suspected to be carried by the migratory birds (Nakao et al., 2021; Terada et al., 2019). *Amblyomma testudinarium* is known as a vector for *R. tamurae*, a causative agent of spotted fever rickettsiosis in humans (Fournier et al., 2006; Imaoka et al., 2011), and SFTS virus in Japan. Recently, a novel thogotovirus, named as Oz virus, was isolated from *A. testudinarium* collected in Ehime prefecture, Japan (Ejiri et al., 2018). Cases of human bites have been reported elsewhere (Isohisa et al., 2011; Nakao et al., 2017). Moreover, the cases of tick‐associated rash illness (TARI) showing manifestation of erythema migrans (EM) without association of Lyme disease have been reported in the patients who were bitten by this tick species (Natsuaki et al., 2014).

High‐throughput sequencing techniques are very functional in species identification and population genetics of ticks (Burger et al., 2012, 2013, 2014). Comparative whole mitochondrial genome (mitogenome) sequence analyses have been used to identify key genetic features among tick species (Kelava et al., 2021; Regilme et al., 2021; Wang et al., 2019). Genetic characterization of complete mitogenome sequences is a core study nowadays due to its highly conserved structure, the high mutation rate, low recombination rate, and maternal inheritance. Recently, two studies reported the complete whole mitogenome of *A. testudinarium*, which enables the comparative analysis in a higher resolution between tick species (Chang et al., 2020; Nakao et al., 2021) than previously implemented intraspecies phylogenetic analyses targeting partial sequences of 16S ribosomal RNA gene (rDNA), 12S rDNA, and cytochrome *c* oxidase I gene (COI) (Chao et al., 2017; Yamauchi et al., 2012).

In the present study, we describe the population structure of *A. testudinarium* based on whole mitogenome sequences obtained from Japan. We investigated the relationships between genetic distances of *A. testudinarium* samples and the corresponding geographical locations from which they were collected. No geographic clustering was detected among *A. testudinarium* sequences from Japan, except the samples from Amami island in Kyushu region. Additionally, we characterized a potentially novel *Amblyomma* species from Myanmar and investigated its genetic relationships with *A. testudinarium* based on the parsimony informative sites of their mitogenomes.

## 2 MATERIAL AND METHODS

### 2.1 Specimen collection and DNA extraction

**FIGURE 1:**
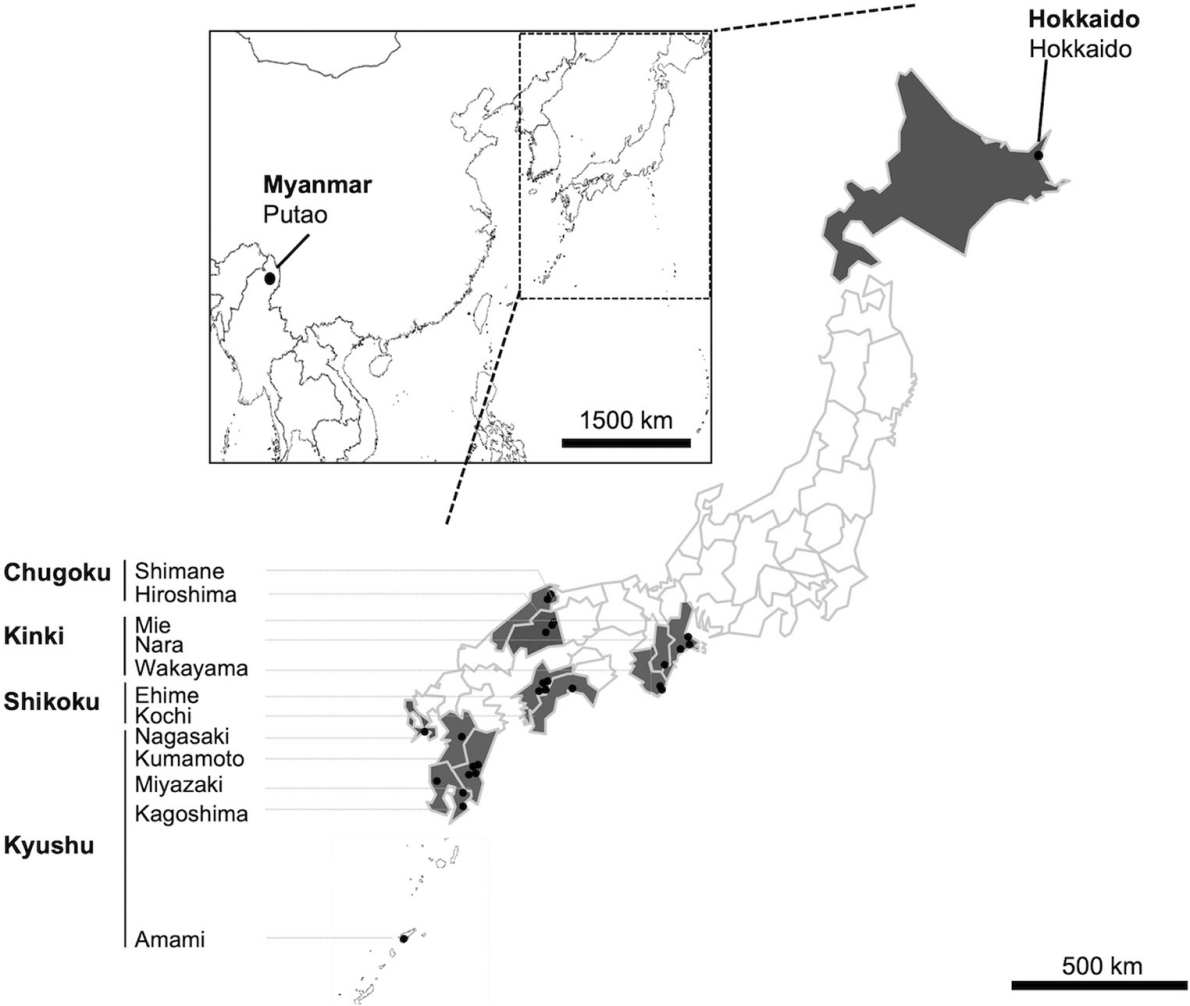
Geographic distribution of *Amblyomma* samples used in the present study. Sample collection sites are illustrated in dots. Samples were collected from eleven prefectures in four geographical blocks in Japan. In each prefecture, samples were collected at least from two different collection sites except for Nagasaki where only one site was explored

A total of 39 tick specimens were included in this study. Thirty ticks were collected from 22 collection sites in four geographical blocks (Kinki, Chugoku, Shikoku, and Kyushu) in Japan and nine specimens from Putao town in Myanmar (Table 1, Figure 1). All tick samples from Japan were collected from the vegetations (*n* = 30), while those from Myanmar were collected from water buffaloes (*Bubalus bubalis*) (*n* = 5) and cattle (*Bos taurus*) (*n* = 4) (Table 1). The collected ticks were kept at 4°C until transferred to the laboratory for further examinations. Species were identified morphologically using standard taxonomic keys (Yamaguti et al., 1971) and a related article (Chamuah et al., 2016) under a stereomicroscope. Then, each tick was cut in half with a sterile stainless‐steel blade. One half was crushed with stainless‐steel beads using a Micro Smash MS‐100R (TOMY, Tokyo, Japan) at 2500 rpm, and the DNA was extracted using a blackPREP Tick DNA/RNA Kit (Analytikjena, Germany) as described previously (Thu et al., 2019).

**TABLE 1:** Geographic origin, collection source, and developmental stage/sex of *Amblyomma* ticks

Geographic block	Prefecture	Collection site	Collection source	Number	Data source	Accession number(s)	Stage/sex
Hokkaido	Hokkaido	Site 1	Brown bear	1	Nakao et al. (2021)	LC553841	Female
Kinki	Mie	Site 1	Vegetation	2	This study	LC554771, LC554772	Nymph
		Site 2		2		LC554773, LC554774	Nymph
		Site 3		2		LC554775, LC554776	Nymph
	Nara	Site 1		1	Kelava et al. (2021)	MT371798	Nymph
	Wakayama	Site 1		1	This study	LC554769	Female
		Site 2		1		LC554770	Female
Chugoku	Hiroshima	Site 1	Vegetation	1	This study	LC554788	Male
		Site 2		1		LC554789	Male
				1		LC554787	Female
	Shimane	Site 1		1		LC554777	Male
		Site 2		1		LC554778	Female
Shikoku	Ehime	Site 1	Vegetation	1	This study	LC554780	Male
		Site 2		2		LC554781, LC554782	Nymph
		Site 3		1		LC554783	Female
	Kochi	Site 1		1		LC554779	Male
Kyushu	Nagasaki	Site 1	Vegetation	1	This study	LC554790	Male
	Kumamoto	Site 1		1		LC554766	Nymph
	Miyazaki	Site 1		2		LC554761, LC554762	Nymph
		Site 2		2		LC554763, LC554764	Nymph
	Kagoshima	Site 1		1		LC554767	Nymph
		Site 2		1		LC554765	Female
		Site 2		1		LC554768	Nymph
	Amami (Kagoshima)	Site 1		1		LC554786	Nymph
		Site 2		2		LC554784, LC554785	Nymph
Myanmar	Putao	Site 1	Cattle	2	This study	LC633546	Male
						LC633547	Female
			Water buffalo	2		LC633548, LC633549	Male
		Site 2	Cattle	1		LC633550	Male
				1		LC633554	Female
			Water buffalo	3		LC633551–LC633553	Male
Total	41						

### 2.2 Next‐generation sequencing and read assembly

The entire mitogenome sequence of *A. testudinarium* was amplified in two PCRs (long‐ and short‐range) according to the previous study with some modifications (Kelava et al., 2021). The long‐range PCR primers (K23A: 5′‐TCCTACATGATCTGAGTTTARACCG‐3′ and ALF3: 5′‐AAATTCCTCTGAAAAGCTTAARRTACC‐3′) and the short‐range PCR primers (A_gap_AMBL_F2: 5′‐AACACTTAACATTTTCATTG‐3′ and A_gap_AMBL_R1: 5′‐ RACHAGGATTAGATACCCTWYTATT‐3′) were designed by aligning complete mitogenomes of genus *Amblyomma* deposited in the database. Long‐range PCR was performed in a 50‐μl reaction mixture containing 10 μl of 5 × PrimeSTAR GXL Buffer (Mg^2+^ Plus) (TaKaRa Bio Inc., Shiga, Japan), 4.0 μl of dNTP Mixture (2.5 mM each), 200 nM of each primer, 1.0 μl of PrimeSTAR® GXL DNA Polymerase (TaKaRa Bio Inc.), and 2.0 μl of template DNA with reaction conditions as follow: 45 cycles of 98°C for 10 s, 60°C for 15 s, and 68 °C for 10 min. Short‐range PCR was performed in a 25‐μl reaction mixture containing 12.5 μl of 2 × Gflex PCR Buffer (Mg^2+^, dNTP plus) (TaKaRa Bio Inc.), 0.5 μl of Tks Gflex DNA Polymerase (1.25 units/μl) (TaKaRa Bio Inc.), 200 nM of each primer, and 1.0 μl of template DNA with the reaction conditions: 94°C for 60 s, 45 cycles of 98°C for 10 s, 55°C for 15 s, 68°C for 60 s, and a final extension of 68°C for 5 min. Electrophoresis in a 1.5% agarose gel stained with Gel‐Red™ (Biotium, Hayward, CA) was used to analyze the amplified PCR products. NucleoSpin Gel and PCR Clean‐Up Kit (TaKaRa Bio Inc.) were used to purify the PCR products.

The long‐range and short‐range PCR products were mixed at equimolar concentrations in the ratio of 7:1, respectively. DNA concentration of mixed PCR products from each sample was adjusted to 0.2 ng/μl. Illumina sequencing libraries were constructed from the purified PCR amplicons with the Nextera DNA Library Prep Kit (Illumina, Hayward, CA). Illumina MiSeq platform was used for sequencing using the MiSeq reagent kit v3 for 600 cycles. CLC Genomics Workbench v20.0.4 (Qiagen, Hilden, Germany) was used to assemble the reads to obtain complete mitogenome sequence of each sample.

### 2.3 Phylogenetic analysis based on 16S rDNA and COI sequences

We constructed the phylogenetic trees using the previously published partial mitochondrial 16S rDNA and COI sequences of *A. testudinarium* samples from Taiwan and Thailand. In addition, we extracted the mitochondrial 16S rDNA and COI sequences from the complete mitogenome sequences of *A. testudinarium* from China, Myanmar, and Japan, six other *Amblyomma* spp., seven *Haemaphysalis* spp., and one each of *Archaeocroton sphenodonti*, *Bothriocroton undatum*, *Dermacentor nitens*, *Dermacentor silvarum*, *Rhipicentor nuttalli*, *Rhipicephalus microplus*, and *Robertsicus elaphensis* available in the database. Sequences of the 16S rDNA were aligned and the best fit model was selected using MEGAX software (Kumar et al., 2018). Maximum‐likelihood (ML) trees were constructed by setting the General Time Reversible (GTR) as a substitution model and gamma distribution at 5 for both the 16S rDNA and COI datasets. The phylogenetic relationship was tested by setting the bootstrap method at 1000 iterations.

Haplotypes were resolved, independently, based on the 16S rDNA sequences extracted from mitogenomes of *A. testudinarium* collected from Japan and the *Amblyomma* sp. from Myanmar using the DnaSP version 6.0 (Librado & Rozas, 2009). Median‐joining network was calculated and constructed with all parameters designated to default values in the Network version 10.2.0.0 (Bandelt et al., 1999) to understand the phylogenetic relationships among haplotypes.

### 2.4 Mitogenome comparison and phylogeny construction

Our complete mitogenome sequences were imported to Geneious version 10.2.6 (Biomatters Ltd., Auckland, New Zealand) and aligned with the mitogenome sequences of *A. testudinarium*, *A. geoemydae*, *Amblyomma javanense*, *Amblyomma fimbriatum*, *Amblyomma triguttatum*, *A. americanum*, *Amblyomma sculptum*, *Haemaphysalis concinna*, *Haemaphysalis formosensis*, *Haemaphysalis flava*, *Haemaphysalis japonica*, *Haemaphysalis hystricis*, *Haemaphysalis longicornis*, *Haemaphysalis inermis*, *A. sphenodonti*, *B. undatum*, *D. nitens*, *D. silvarum*, *R. nuttalli*, *R. microplus*, and *R. elaphensis*. A total of 13 protein coding gene (PCG) and two rDNA sequences were extracted from each mitogenome sequence and concatenated together.

Sequences of the concatenated 15 mitochondrial genes of *Amblyomma* samples collected from Japan and Myanmar along with those obtained from the GenBank were aligned using MAFFT software (Katoh & Standley, 2013). A substitution model was selected using PHYML 3.0 software relying on the Akaike Information Criterion (Guindon et al., 2010). A pairwise identity analysis was conducted by calculating the percentage of residues that are identical between sequences in Geneious software.

Bayesian phylogenetic tree was constructed using BEAST version 1.4. as a cross‐platform program for Bayesian analysis of molecular sequences using Markov chain Monte Carlo (MCMC). We modeled the evolution of sequences by using the GTR nucleotide substitution model with discrete gamma‐distributed rate variation and assuming a constant evolution rate all over the tree by selecting the strict clock model. We assume that the Bayesian skyline coalescent model was a demographic model in a Bayesian framework. The Bayesian skyline plot analysis was performed using a chain length of 50 million generations sampled every 50,000 MCMC steps with a pre‐burn‐in of 500,000. Maximum clade credibility (MCC) was selected by using the TreeAnnotator (Drummond & Rambaut, 2007). The MCC tree was illustrated using FigTree version 1.4.4 (http://beast.bio.ed.ac.uk/figtree), and the branch length of the tree was transformed to proportional and supported by the posterior values. To estimate the differences between *A. testudinarium* sequences in relation to the geographical differences, we performed a nonmetric multidimensional scaling (NMDS) analysis and visualized the results in space using the R package Bios2mds (Pele et al., 2012).

### 2.5 Population genetic structure analyses

The analysis of molecular variance (AMOVA) (Excoffier et al., 2007) in Arlequin software version 3.5.2.2 was used to evaluate the genetic variance among and within the populations of *A. testudinarium* collected from Amami island in Kyushu region vs other localities in Japan. We adjusted the number of permutations at 1000, and the difference was considered significant when tested at *p* < 0.05 level based on the calculated fixation indices (*F*‐statistics). *F*_ST_ estimates the degree of differentiation within the population where the closer *F*_ST_ is to 0, the greater the extent of allelic fixation or identity within populations (Holsinger & Weir, 2009). *F*_SC_ estimates the differentiation among populations within the group to which they are assigned. The closer *F*_SC_ is to 1, the more heterogeneity among populations exists. In case a strong population genetic structure exists at the population scale being analyzed, *F*_SC_ should be high relative to *F*_ST_.

### 2.6 Spatial and demographic evolutionary dynamics analyses

The historical expansion dynamics of *A. testudinarium* populations were estimated by calculating the mismatch distribution based on 15 concatenated mitochondrial genes in Arlequin software (Rogers & Harpending, 1992). We compared the observed and expected distributions of pairwise nucleotide variations between individuals. Multimodal distribution is thought to be associated with a constant population size, whereas unimodal distribution is representing a sudden expansion. The deviation of observed mismatch from the individual expansion model was assessed by estimating the sum of square deviation (SSD); however, the Harpending’s raggedness index (RI) was applied to assess the smoothness of the haplotype frequency distribution (Harpending, 1994) where significant RI indicates a poor fit of the data to the expansion model.

Departure from the neutral model of evolution was evaluated through calculating Tajima’s *D* statistics (Tajima, 1989) in the Arlequin software based on the infinite‐size model by quantifying the difference between the average pairwise nucleotide differences and polymorphic sites. Significantly negative value of Tajima’s *D* suggests a positive selection or exhibiting a sudden population expansion after a recent bottleneck event. Significantly positive value of Tajima’s *D* means an increase of average pairwise genetic diversity in a population, indicating a balancing selection model or a population contraction events such as population subdivision. Nonsignificant and near zero values of Tajima’s *D* mean a constant population size.

Mantel test was used to test whether there is a geographic separation between *A. testudinarium* populations from different geographic origins and to evaluate the correlation between genetic variation and geographic distance of the tick population. R function “*mantel.rtest*” in ape package was used (Wynn et al., 2016). Scatter plot of the correlation was created using the R package ggplot2 (Ginestet, 2011; Villanueva & Chen, 2019).

### 2.7 Data accessibility

The complete mitogenome sequences of 30 *A. testudinarium* and 9 *Amblyomma* sp. were deposited in the DNA Data Bank of Japan (http://www.ddbj.nig.ac.jp) with accession numbers of LC554761‐LC554790 (samples from Japan) and LC633546‐LC633554 (samples from Myanmar).

## 3 RESULTS

### 3.1 Features of the sequenced mitochondrial genomes

The length of assembled mitogenomes from 30 *Amblyomma* specimens from Japan and 9 from Myanmar ranged from 14,830 to 14,839 bp. A total of 37 encoded genes were detected including 13 PCGs, 22 transfer RNA (tRNA) genes, and two rDNAs. Two control regions were identified in the mitogenome. There was no difference in gene rearrangement among the 39 newly sequenced *Amblyomma* mitogenomes.

### 3.2 Nucleotide identities based complete mitogenome sequences

Generated nucleotide sequences of 30 collected ticks from Japan and 9 from Myanmar were aligned with the reference mitogenome sequences of *A. testudinarium* from Japan (accession number: MT371798) along with six *Amblyomma* spp. and 14 other ixodid ticks including seven *Haemaphysalis* spp., one each of *A. sphenodonti*, *B. undatum*, *D. nitens*, *D. silvarum*, *R. nuttalli*, *R. microplus*, and *R. elaphensis*. Comparative molecular identity analysis of the assembled nucleotide sequences from Japan and Myanmar showed an average nucleotide identity of 74.72% (71.00–91.48) and 74.56% (71.23–79.55) with the other 20 ixodid species, respectively (Table 2). The average nucleotide identity of mitogenome sequences from Japan was 99.80% with the reference mitogenome sequence of *A. testudinarium* (accession number: MT371798) collected from Nara, Japan. The average nucleotide identity of the mitogenome sequences from Myanmar was 91.32% with the same reference mitogenome of *A. testudinarium* (accession number: MT371798) (Table 2). In addition, the average nucleotide identities of *Amblyomma* mitogenome sequences from Japan and Myanmar were, respectively, 79.50% and 79.28% to *A. testudinarium* from China (accession number: MT029329) and 78.45% and 78.37% to *A. javanense* (accession number: NC_043872) (Table 2).

**TABLE 2:** Summary of the molecular identity of *Amblyomma* mitogenomes

Comparison (accession number)	Average identity (%) (min–max)
*A. testudinarium* from Japan vs other ixodid species^a^	74.72 (71.00–91.48)
*Amblyomma* sp. from Myanmar vs other ixodid species^a^	74.56 (71.23–79.55)
*A. testudinarium* from Japan vs *A. testudinarium* Nara strain (MT371798)	99.80 (99.34–99.93)
*Amblyomma* sp. from Myanmar vs *A. testudinarium* Nara strain (MT371798)	91.32 (91.40–91.46)
*A. testudinarium* from Japan vs *A. javanense* (NC_043872)	78.45 (79.63–79.74)
*Amblyomma* sp. from Myanmar vs *A. javanense* (NC_043872)	78.37 (77.16–79.63)
*A. testudinarium* from Japan vs *A. testudinarium* from China (MT029329)	79.50 (79.48–79.56)
*Amblyomma* sp. from Myanmar vs *A. testudinarium* from China (MT029329)	79.28 (79.26–79.32)
*A. testudinarium* from China (MT029329) vs *A. javanense* (NC_043872)	83.72 (81.43–86.02)

^a^ A total of 20 ixodid species (*A. javanense*, *A. geoemydae*, *A. fimbriatum*, *A. triguttatum*, *A. americanum*, *A. sculptum*, *H. concinna*, *H. formosensis*, *H. flava*, *H. japonica*, *H. hystricis*, *H. longicornis*, *H. inermis*, *A. sphenodonti*, *B. undatum*, *D. nitens*, *D. silvarum*, *R. nuttalli*, *R. microplus*, and *R. elaphensis*) were used to compare *Amblyomma* mitogenomes in the study.

### 3.3 Phylogenetic relationship based on mitochondrial 16S rDNA and COI

**FIGURE 2:**
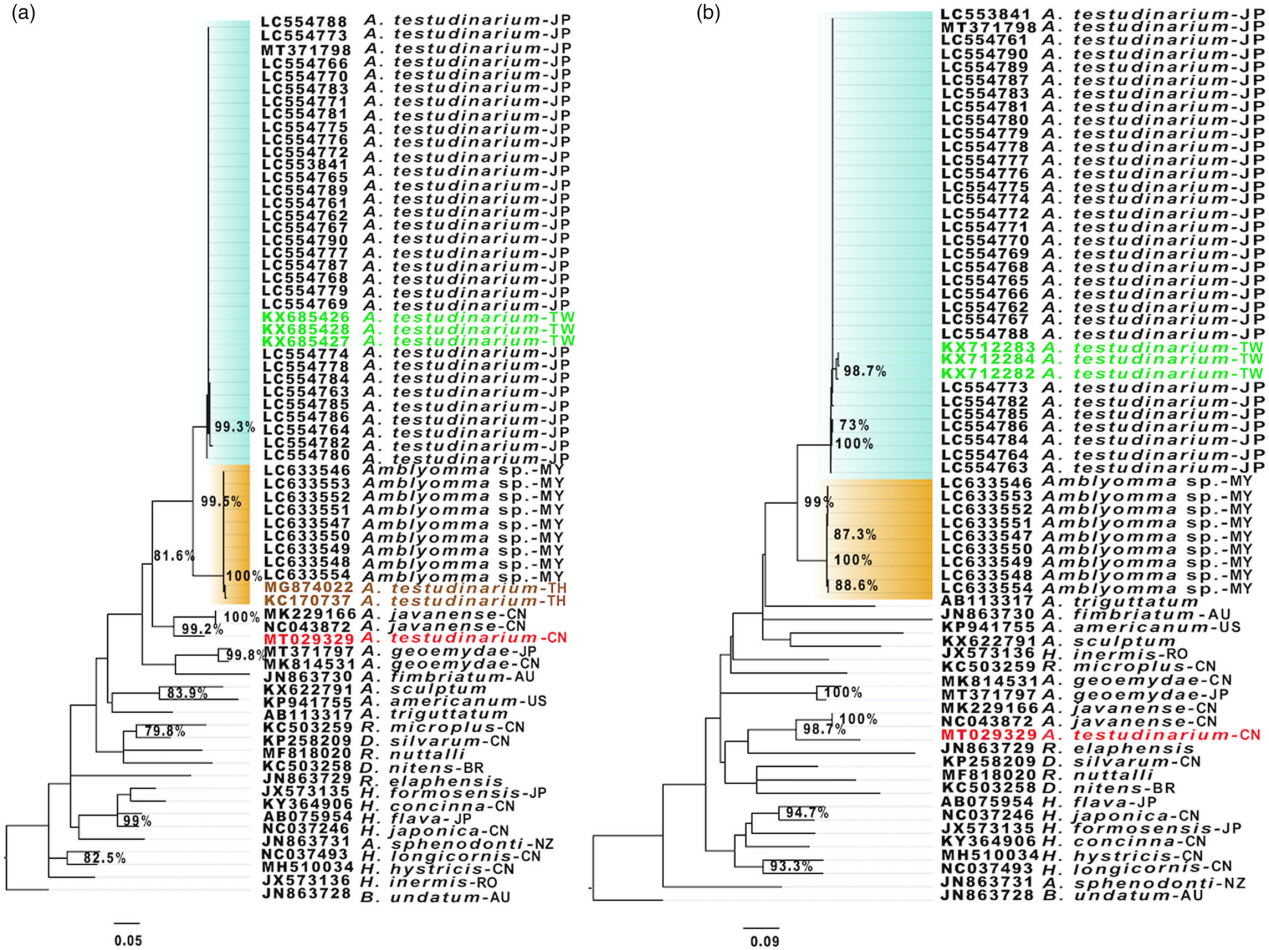
Maximum‐likelihood trees based on the mitochondrial (a) 16S rDNA and (b) COI gene. Labels in green, brown, and red indicate the sequences from Taiwan, Thailand, and China, respectively. The clade containing sequences from Japan and Taiwan is highlighted in cyan and the clade highlighted in orange represents the clade containing sequences from Myanmar. Abbreviations in the sample name refers to the country/continent of origin; AU, Australia; BR, Brazil; CN, China; JP, Japan; MY, Myanmar; NZ, New Zealand; TH, Thailand; TW, Taiwan; RO, Romania; US, USA. Bootstrap values above 70% are indicated by percentages

To investigate the phylogeographic relationships of *Amblyomma* spp., we constructed a phylogenetic tree based on the 16S rDNA sequences. The constructed ML tree showed that *A. testudinarium* sequences from Japan and Taiwan clustered together in one clade and were closely related to another clade containing the sequences from *Amblyomma* sp. collected from Myanmar and *A. testudinarium* from Thailand (Figure 2). Interestingly, *A. testudinarium* sequence from China (accession number: MT029329) did not cluster with either the *A. testudinarium* from Japan or *Amblyomma* sp. sequences from Myanmar but formed a separate cluster with *A. javanense* sequences from China (accession numbers: NC_043872 and MK229166). The topology of the phylogenetic trees suggested that the sequence identified as *A. testudinarium* in China (accession number: MT029329) is different from the other *A. testudinarium* sequences from Japan and Taiwan (Figure 2). In addition, the phylogenetic analysis of *A. testudinarium* based on the COI sequences was similar to the results of the 16S rDNA where the sequences of *A. testudinarium* from Japan and Taiwan clustered in a monophyletic group. *Amblyomma* sp. sequences from Myanmar formed a separate clade in both 16S rDNA and COI‐based phylogenetic trees. The COI sequences of *A. testudinarium* from China (accession number: MT029329) clustered with *A. javanense* but not with other *A. testudinarium* sequences from other Asian countries (Figure 2).

### 3.4 Phylogeographic analysis based on the concatenated 13 PCGs and two rDNAs

**FIGURE 3:**
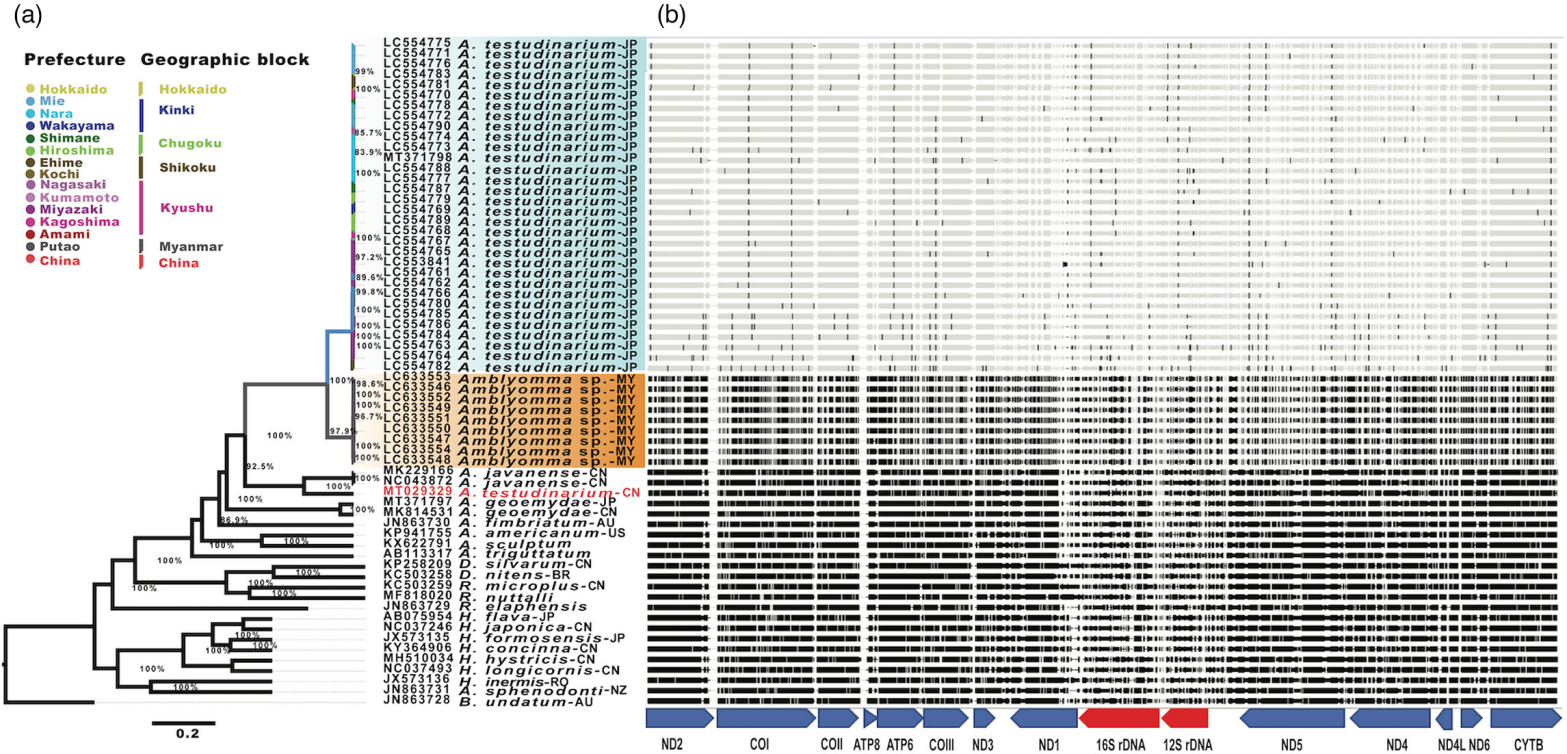
Genetic diversity and phylogenetic relationships among *Amblyomma testudinarium* from Japan and *Amblyomma* sp. from Myanmar. (a) Bayesian phylogenetic Maximum Clade Credibility (MCC) tree based on the 15 concatenated mitochondrial gene sequences. The tree was rooted to *Bothriocroton undatum* (accession number: JN863728). Clades of the specimen from Japan and Myanmar are heighted in cyan and orange, respectively. Branch colors represent sampling geographic origin of each sequence. (b) The nucleotide differences between 39 new mitogenome sequences of *Amblyomma* collected from different localities in Japan and Myanmar. Locations of single nucleotide variations are indicated as vertical lines in mitogenome sequences relative to the mitogenome sequence of *A. testudinarium* Nara strain (accession number: MT371798). Abbreviations in the sample names refer to the country of origin; AU, Australia; BR, Brazil; CN, China; JP, Japan; MY, Myanmar; NZ, New Zealand; RO, Romania; US, USA

A phylogeographic tree was constructed with a set of 13 PCGs concatenated with two rDNAs extracted from the complete mitogenomes of 32 *A. testudinarium* specimens collected from eleven prefectures in Japan, nine *Amblyomma* sp. from Putao in Myanmar, and 19 Ixodidae species from the GenBank including six *Amblyomma* spp., seven *Haemaphysalis* spp., and one each of *A. sphenodonti*, *B. undatum*, *D. nitens*, *D. silvarum*, *R. nuttalli*, *R. microplus*, and *R. elaphensis*. The topology of the constructed MCC showed agreement with the constructed ML tree based on the 16S rDNA and COI in that a total of 32 *A. testudinarium* sequences from Japan clustered together and nine *Amblyomma* sp. sequences from Myanmar formed a separate cluster (Figure 3). The sequences of *A. testudinarium* and *Amblyomma* sp. were closely related to *A. javanense* and *A. geoemydae*. Most of the tree branches showed strong bootstrap support values.

### 3.5 Haplotyping based on the 16S rDNA of *A. testudinarium* from Japan

**FIGURE 4:**
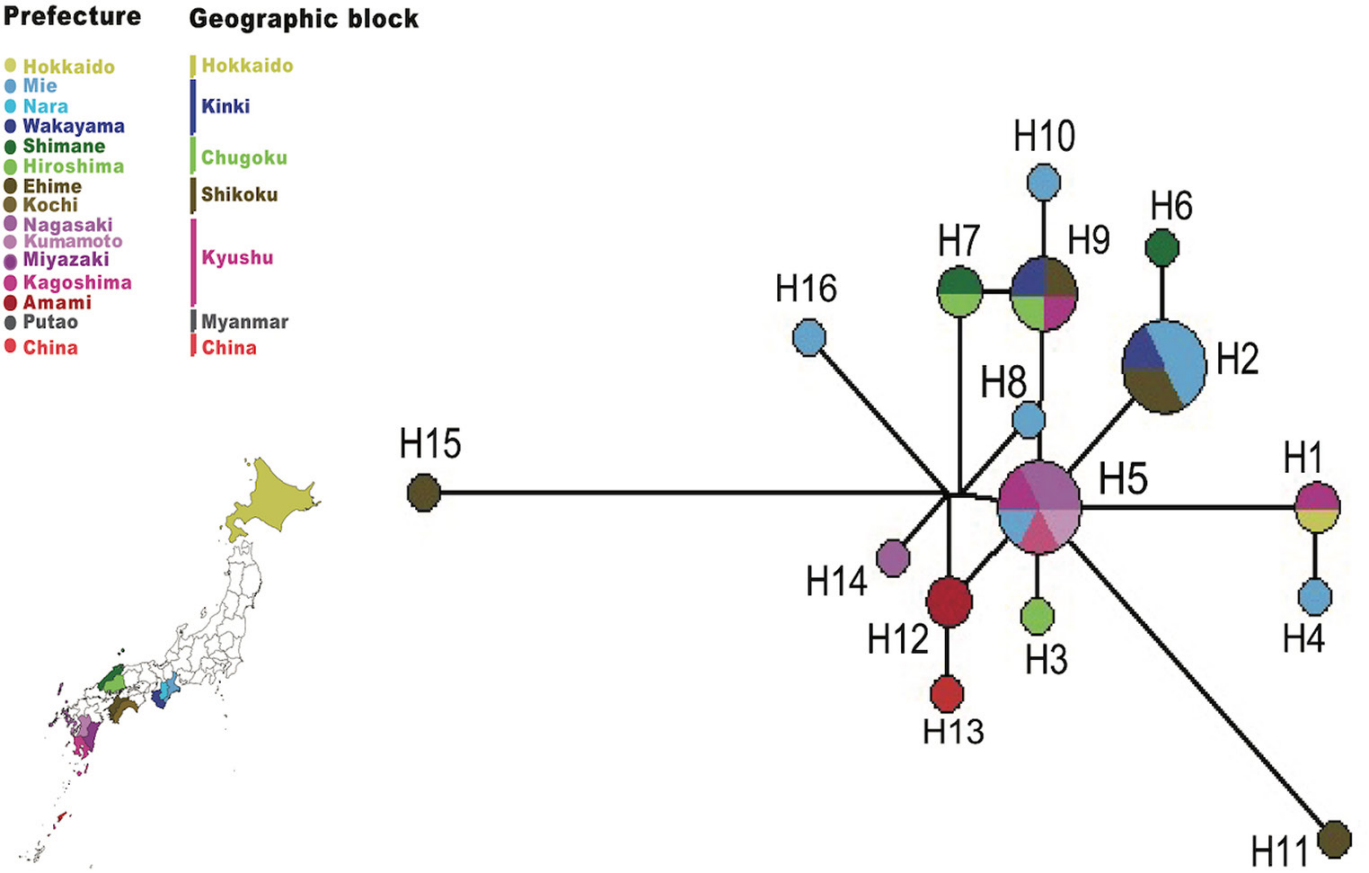
Reduced‐median‐joining phylogenetic network of the 16 haplotypes identified based on the 16S rDNA sequences. Colors in the pie represent the geographical origin of each haplotype. Node size is proportional to the number of individuals

To understand the genetic population structure of *A. testudinarium* in Japan, we performed a haplotype analysis based on 32 16S rDNA sequences from Japan. The haplotype analysis resolved a total of 16 haplotypes, which were defined by 28 single nucleotide polymorphic sites (SNPs). Median‐joining phylogenetic network analysis for the 16 haplotypes revealed that the *A. testudinarium* population from Japan forms a star‐like phylogeny, with the common ancestorial haplotype (H5) in the center surrounded by the remaining haplotypes (Figure 4). The most frequent three haplotypes in the population from Japan were H2, H5, and H9 that were observed in six, six, and four samples, respectively. A total of five (H1, H2, H5, H7, and H9) were shared between different sampling sites (Figure 4).

### 3.6 Population structure of *A. testudinarium*

**FIGURE 5:**
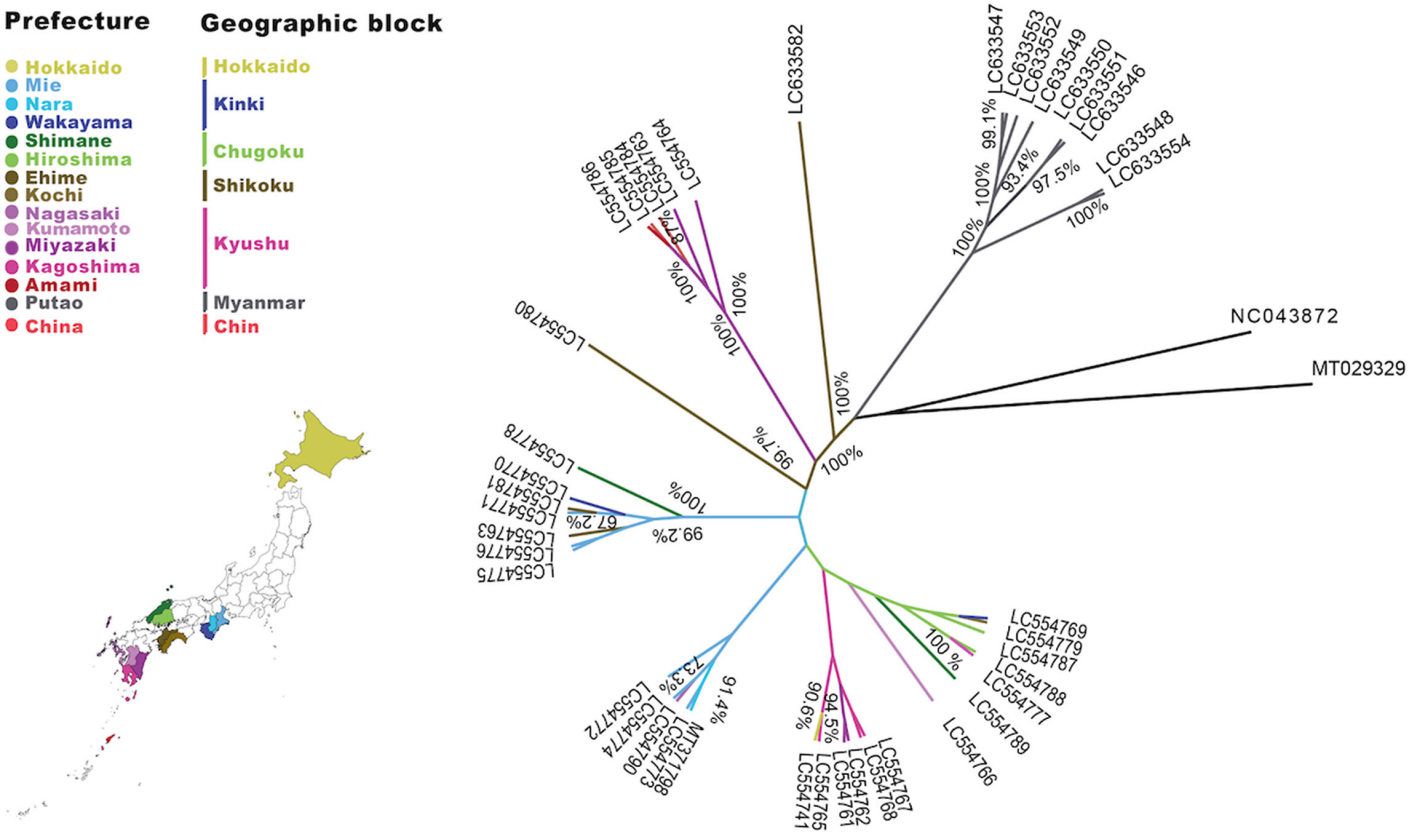
A maximum clade credibility (MCC) tree based on Bayesian approach. Tree branches are colored based on the geographic regions of sample collection sites. Tree was rooted to *Amblyomma testudinarium* (MT029329) and *Amblyomma javanense* (NC_043872) reported from China (branches are shown in bold)

The phylogeographic analysis based on the 15 concatenated mitochondrial genes extracted from 32 *A. testudinarium* from Japan and 9 *Amblyomma* sp. from Myanmar, where *A. javanense* was used as an outgroup, showed the separation of Japan population of *A. testudinarium* from the *Amblyomma* sp. sequences from Myanmar. Interestingly, *A. testudinarium* sequences from Amami island and two sequences from Miyazaki prefecture were grouped and separated from the remaining Japan populations (Figure 5). In addition, one *A. testudinarium* sequence from Ehime prefecture was separated from the remaining sequences from Japan.

**FIGURE 6:**
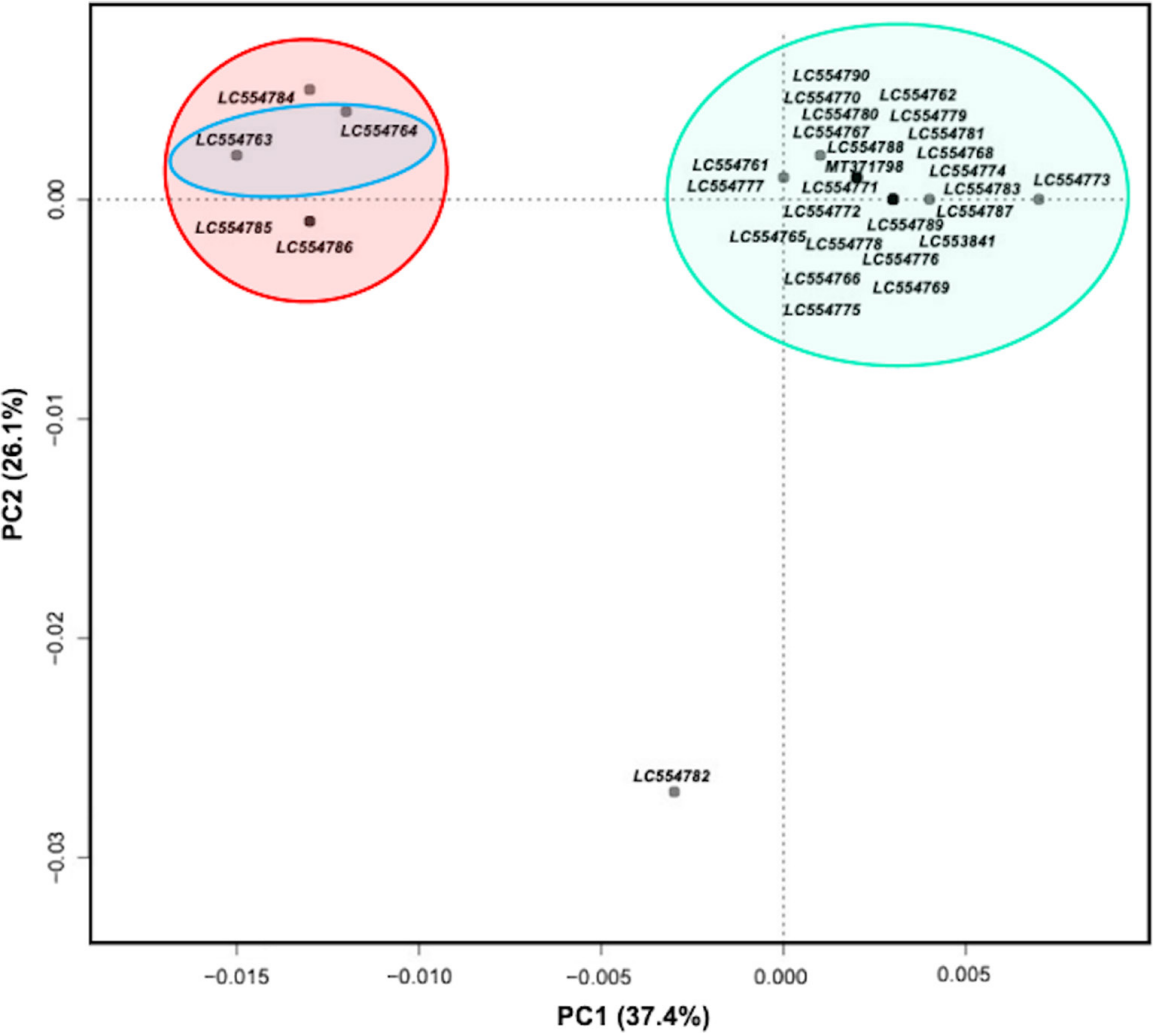
Nonmetric multidimensional scaling (NMDS) ordination plot based on the distance matrix. Red, blue, and cyan ellipsoids represent the grouping of *Amblyomma testudinarium* from Amami, Miyazaki (two samples collected at site 2), and the remaining Japan localities, respectively

Analysis of percentile identity based on the concatenated 15 mitochondrial genes of *A. testudinarium* used in the phylogeny revealed that three sequences of *A. testudinarium* from Amami clustered with two sequences from Miyazaki and showed 99.89% identity with each other, 99.66% to other samples from Miyazaki, and 99.51% to the remaining sequences from Japan. The average identity of *A. testudinarium* from Ehime was 99.66% (99.27–99.98) to the other sequences from Japan. The average diversity between *A. testudinarium* sequences from Japan and *Amblyomma* sp. sequences from Myanmar was 8.52%, showing 8.43% to 8.65% in comparison to the reference mitogenome *A. testudinarium* (accession number: MT371798) from Japan. Comparison of variation at different PCGs indicated that ATP8, ATP6, and ND2 had the highest diversity among the 15 mitochondrial genes (Table 3 and Figure 3b). In addition, the molecular diversity indices showed that *A. testudinarium* population in Japan is more genetically diverse than the *Amblyomma* sp. of Myanmar (Table S1). In addition, the nonmetric multidimension scale analysis based on the genetic distances confirmed the geographic separation and genetic distinction of *A. testurinarium* from Amami island and Miyazaki (two samples collected at site 2), which were clustered together in one group and separated from other samples from Japan (Figure 6). We also showed the genetic distinction and possible geographical separation of *A. testurinarium* from Ehime prefecture based on the NMDS analysis.

**TABLE 3:** Comparison of variation at different PCGs for the *Amblyomma testudinarium* samples

Gene name	Number of haplotypes	Nucleotide length (min–max)	Gene product	GC content (%)	Polymorphic sites (%)	Parsimony informative sites (%)
12S rDNA	14	715(711–723)	12S ribosomal RNA	18.1	19.7 (141/715)^a^	8.0 (57/715)^b^
16S rDNA	16	1208 (1202–1223)	16S ribosomal RNA	16.8	20.3 (245/1208)	8.7 (105/1208)
**ATP6**	10	663 (663‐663)	ATP synthase subunit 6	18.4	23.7 (157/663)	**10.1 (67/663)**
**ATP8**	4	159 (159–162)	ATP synthase subunit 8	12.7	35.8 (57/159)	**17.0 (27/159)**
COI	16	1538 (1536–1542)	Cytochrome *c* oxidase subunit I	27.1	18.5 (285/1538)	7.9 (122/1538)
COII	11	675 (675–675)	Cytochrome *c* oxidase subunit II	24.0	22.1 (149/675)	7.9 (53/675)
COIII	14	778 (778–778)	Cytochrome *c* oxidase subunit III	22.5	20.8 (162/778)	5.8 (45/778)
CYTB	19	1077 (1077–1077)	Cytochrome b	21.5	22.7 (245/1077)	6.5 (70/1077)
ND1	8	1010 (1007–1023)	NADH dehydrogenase subunit 1	18.8	22.2 (224/1010)	7.2 (73/1010)
**ND2**	13	963 (963–963)	NADH dehydrogenase subunit 2	13.2	27.0 (260/963)	**9.9 (95/963)**
ND3	6	342 (342–342)	NADH dehydrogenase subunit 3	14.0	23.7 (81/342)	9.4 (32/342)
ND4	21	1326 (1326–1326)	NADH dehydrogenase subunit 4	18.7	26.0 (345/1326)	9.0 (119/1326)
ND4L	6	276 (276–276)	NADH dehydrogenase subunit 4L	14.2	21.0 (58/276)	7.2 (20/276)
ND5	23	1656 (1656–1656)	NADH dehydrogenase subunit 5	16.8	24.2 (400/1656)	7.4 (122/1656)
ND6	6	432 (429–432)	NADH dehydrogenase subunit 6	12.5	30.1 (130/432)	8.8 (38/432)

*Note*: The three genes showing the highest diversity levels are shown in Bold.

^a^ Number of polymorphic sites/average number of base pairs.

^b^ Number of parsimony informative sites/average number of base pairs.

**FIGURE 7:**
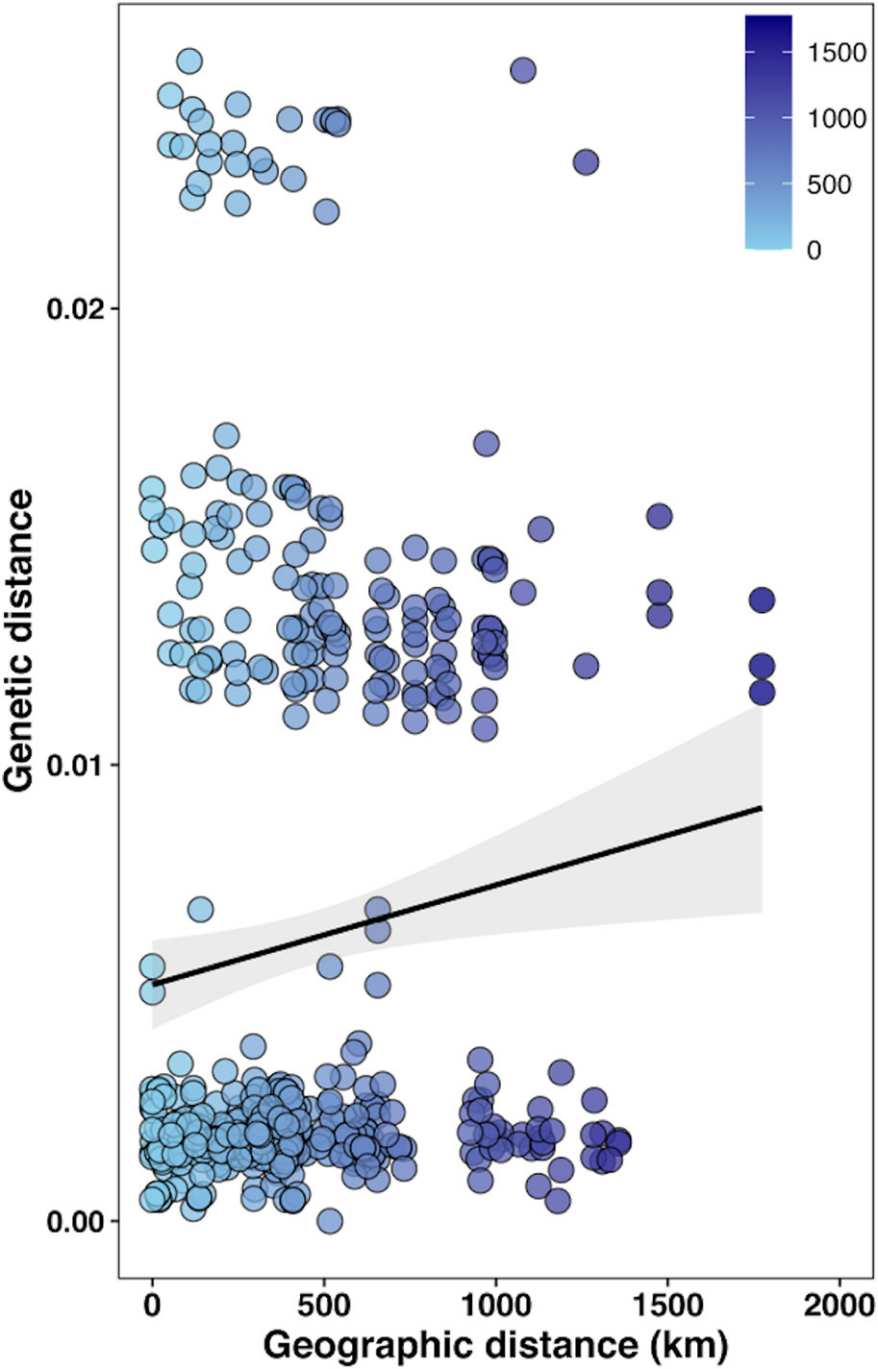
Scatter plot of genetic variation versus geographic distances in kilometer (km) between the four sampling regions in Japan. Regression line is overlayed

To statistically test the population genetic structure between and within populations, we used AMOVA to determine whether the genetic structure was influenced by the geographic structure. Molecular variance results revealed that the genetic variation among‐population of *A. testudinarium* from Amami island (Kyushu) and the other regions in Japan indicated that the population of *A. testudinarium* is structured with one population present only in Amami (Kyushu), where the among‐population genetic variation (57.55%) was higher than that within‐population (42.45%). Significant global *F*_ST_ values (*p* < 0.01) indicated significant genetic differentiation between populations from Amami and the remaining localities (*p* = 0.00098) (Table 4). The correlation analysis of *A. testudinarium* from Japan using Mantel test revealed that there is no significant correlation between the degree of genetic variation based on the pairwise genetic distances and geographic distances with r statistics = 0.11 (*p*‐value = 0.1779 based on 9999 replicates) (Figure 7).

**TABLE 4:** Analysis of molecular variance (AMOVA) using the concatenated 15 mitochondrial genes sequences extracted from whole mitogenomes of *Amblyomma testudinarium* populations in Japan

Populations	Source of variation	Degree of freedom	Sum of squares	Variance components	Percentage of variation	*F*_ST_
Amami island Other regions (Japan)	Among populations	1	71.48	11.57	57.55	57.58^***^
	Within populations	30	256.15	8.54	42.45	

*Note*: Degree of significance: *p < 0.05, **p < 0.01, ***p < 0.0.

**FIGURE 8:**
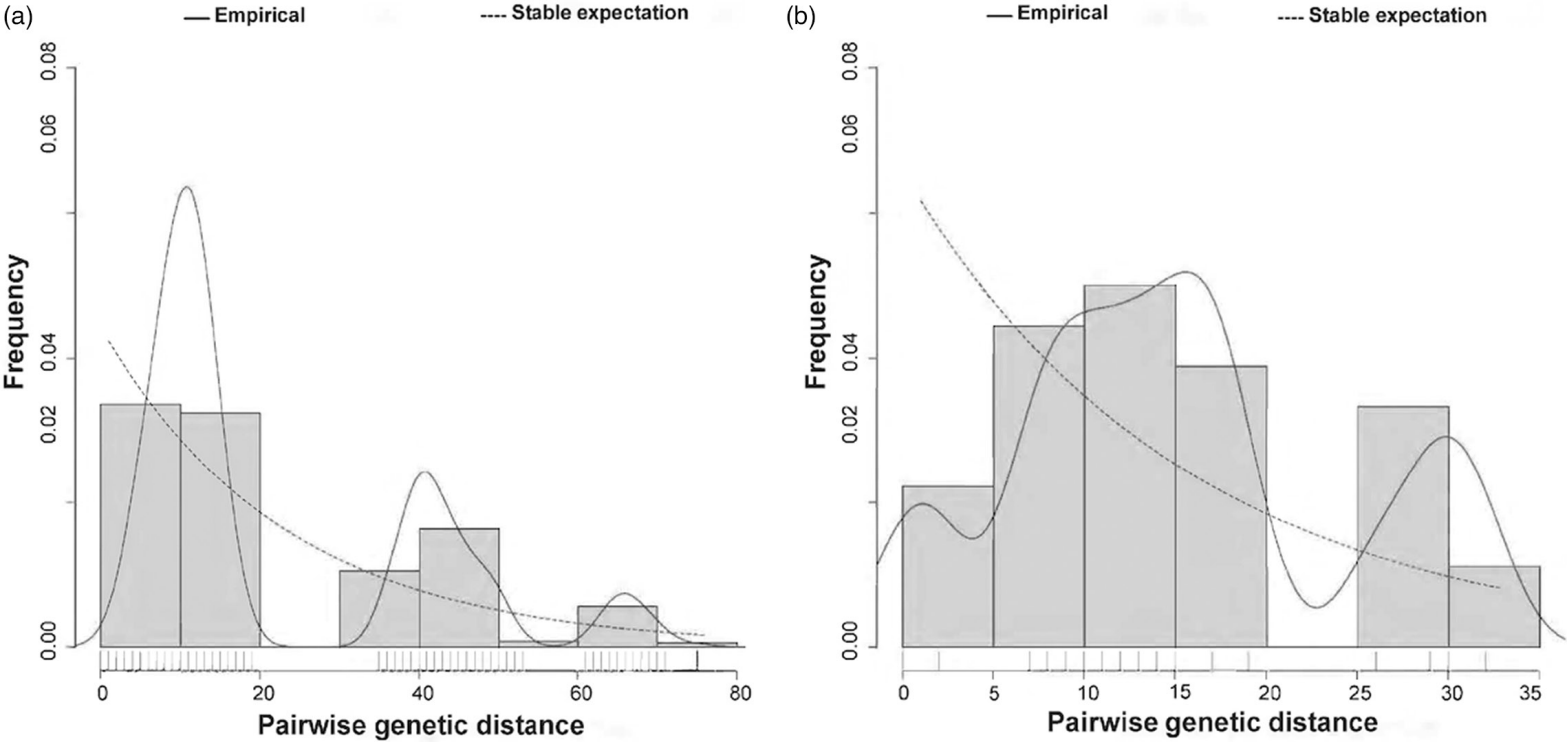
Mismatch distribution pattern for *Amblyomma testudinarium* from Japan and *Amblyomma* sp. from Myanmar based on the 15 concatenated mitochondrial gene sequences. (a) Mismatch distribution pattern for *A. testudinarium* sequences from Japan. (b) Mismatch distribution pattern for *Amblyomma* sp. sequences from Myanmar. The x‐axis shows the number of pairwise differences (genetic distance) between pairs of sequences and the y‐axis shows their frequency. Solid histograms illustrate the observed frequencies. Solid black line indicates the simulated mismatch distributions expected under demographic expansion and dotted black line indicates those expected under spatial expansion

Results of the mismatch analysis based on pairwise nucleotide differences between 41 nucleotide sequences from Japan (*n* = 32) and Myanmar (*n* = 9) revealed that the shape of the mismatch distributions is far from unimodal distribution while analyzing either sequences of *A. testudinarium* from Japan or *Amblyomma* sp. from Myanmar, suggesting a possibility of long‐term demographic stability in each with a constant population size (Figure 8). To determine whether the observed mismatch deviated significantly from a population expansion model, we estimated the SSD and RI for the spatial and demographic expansion model. The observed mismatch pattern of *A. testudinarium* from Japan did not deviate significantly from that expected under population expansion scenario (SSD = 0.015; *p* = 0.180 and *p* = 0.160 for demographic and spatial expansion, respectively), and the similar result was observed for the *Amblyomma* sp. population from Myanmar (SSD = 0.033; *p* = 0.47 and *p* = 0.69 for demographic and spatial expansion, respectively). In addition, the *RI* index for *A. testudinarium* haplotypes in Japan was 0.008 (*p* = 0.49 and *p* = 0.63 for the demographic and spatial expansion model, respectively) and the one for *Amblyomma* sp. in Myanmar was 0.062 (*p* = 0.51 and *p* = 0.73 for the demographic and spatial expansion model, respectively). The results of SSD and RI indices suggested a sudden expansion of *A. testudinarium* in Japan and a stability of the population size of *Amblyomma* sp. population in Myanmar (Table 5).

**TABLE 5:** Evolutionary dynamics of *Amblyomma testudinarium* from Japan and *Amblyomma* sp. from Myanmar identified from 15 concatenated mitochondrial gene sequences

Statistics	Japan	Myanmar	Mean	SD
Spatial expansion				
SSD	0.015	0.033	0.016	0.016
Model (SSD) *p* value	0.160	0.690	0.283	0.361
RI	0.008	0.062	0.023	0.033
Model (RI) *p* value	0.630	0.730	0.453	0.395
Demographic expansion				
SSD	0.015	0.033	0.016	0.016
Model (SSD) *p* value	0.180	0.470	0.216	0.237
RI	0.009	0.062	0.023	0.033
Model (RI) *p* value	0.490	0.510	0.333	0.288

Abbreviations: SSD, sum of square deviation; RI, Harpending’s raggedness index; SD, standard deviation.

Tajima’s *D* test assessing departure from the neutral evolutionary model based on the 15 concatenated mitochondrial genes of *A. testudinarium* and *Amblyomma* sp. showed that the Tajima’s *D* value was calculated to be ‐2.26 in Japan population and 0.06 for the *Amblyomma* sp. from Myanmar. Tajima’s D value was significant in Japan population (*p*‐value = 0.0), suggesting a possibility of a recent population expansion after a bottleneck; however, Tajima’s *D* value was not significant and near zero in the Myanmar population, indicating that the mitogenome does not reject the neutral model hypothesis (Table 6). This suggests that *Amblyomma* sp. in Myanmar might have experienced a weak signal of population bottleneck that reduced its diversity, or this group is characterized by a constant population size (demographic equilibrium), which could happened as the samples in Myanmar were collected from a single collection site.

**TABLE 6:** Neutrality test values of *Amblyomma testudinarium* from Japan and *Amblyomma* sp. from Myanmar based on the concatenated 15 mitochondrial gene sequences

Statistics	Japan	Myanmar	Mean	SD
Tajima's *D* test				
Sample size	32.00	9.00	20.50	16.26
S	207.00	37.00	122.00	120.21
Pi	21.14	13.78	17.46	5.20
Tajima's *D*	−2.26	0.06	−1.10	1.64
Tajima's *D p* value	0.00	0.54	0.27	0.38

Abbreviations: S, singleton sites; Pi, parsimony informative sites; SD, standard deviation.

## 4 DISCUSSION

Phylogeographic structure of tick populations can help enrich the knowledge on the distribution of ticks and tick‐borne pathogens (Black et al., 2001). In addition, intraspecies genetic differences within geographically distinct populations of ticks has been well established (Burnard & Shao, 2019; Mechai et al., 2013; Sakamoto et al., 2014). The current work is the first to assess the genetic diversity and population structure of *A. testudinarium*, which is one of the most widely distributed *Amblyomma* in East and Southeast Asia (Voltzit, 2002) and one of the main ticks that bite humans in Japan (Natsuaki, 2021). Our study is the second to use whole mitogenomes of multiple individuals of the same tick species to assess the genetic diversity based on the regional scale after the report on *Ixodes ricinus* in Italy (Carpi et al., 2016). Although the earlier study detected intraspecies genetic relationships, population differentiation, and demographic history of *I. ricinus*, our study provides an advantage of the wide spatial scale over which our samples were collected and phylogenetic tree reconstruction using 30 new *A. testudinarium* mitogenome sequences as well molecular characterization of a potentially novel *Amblyomma* sp. closely related to *A. testudinarium* based on nine mitogenome sequences from Myanmar.

Only a few studies have focused on ticks in Myanmar (Petney et al., 2019) including *A. testudinarium*, which was reported from other countries in Southeast Asia. Taxonomy of the genus *Amblyomma* is currently under revision and several species were re‐assigned to a new subfamily Bothriocrotoninae (Klompen et al., 2002) or to new genera *Archaeocroton* and *Robertsicus* (Barker & Burger, 2018; Hornok et al., 2020). The species identification of *Amblyomma* species collected in Myanmar is hampered by the lack of reference genomic sequence data for many *Amblyomma* spp. including *A. testudinarium* as well as the lack of morphological description of *A. testudinarium* from Myanmar. Although *A. testudinarium* has been considered widespread and common in East and Southeast Asia, our study suggested that potentially several cryptic *Amblyomma* spp. closely related to *A. testudinarium* may be distributed in Myanmar, Thailand, and China.

Mitogenomes have been used as a marker in population genetics, phylogeography, and DNA barcoding (Eimanifar et al., 2018; Fourdrilis et al., 2016). Of note, mitogenomes were proven to provide a better opportunity to understand tick taxonomy than targeting only a single gene (Kelava et al., 2021). Previously, intraspecies phylogenetic relationships was investigated through targeting the partial sequences of 16S rDNA, 12S rDNA, and COI (Chao et al., 2017; Yamauchi et al., 2012). Since different genes have different evolutionary rates, the final conclusions could have been affected by the targeted gene topology of the phylogenetic tree (Liu et al., 2018). Traditionally, morphological characterization has been used for species identification and differentiation of *Amblyomma* ticks (Aguilar‐Dominguez et al., 2021; Namgyal et al., 2021). Now, a molecular based characterization can provide insights on the genetic variance at the base‐pair level and the genetic diversity between and within species of *Amblyomma* ticks (Burger et al., 2012; Lopes et al., 2016). An important advantage of targeting tick mitogenomes to study its population genetics and distribution is the significantly smaller size when compared to nuclear genomes (Nadolny et al., 2016), which enables the cost‐effective comparative genome analysis. We observed that the gene order of the mitogenome of *A. testudinarium* from Japan and *Amblyomma* sp. from Myanmar was identical to that of the other members of genus *Amblyomma*, which confirms that gene order and composition are highly conserved between *Amblyomma* species to a common ancestor (Chauve & Tannier, 2008; de Lima et al., 2017; Namgyal et al., 2021).

Although we examined tick specimens collected from six different biogeographic regions in Japan with different ecoclimatic habitats, phylogenetic analysis revealed that all sequences from Japan clustered in a monophyletic group with percentile identity of 99.36–100%. However, we demonstrated that mitogenomes derived from *A. testudinarium* in Japan and *Amblyomma* sp. ticks from Myanmar are genetically distinguished from each other and were assigned to a biphyletic group with sequence identity range of 91.35–91.57%. These results are supported by a previous report (Nakao et al., 2021), where the mitogenome of *A. testudinarium* from Hokkaido, the northernmost main island of Japan, was clustered with the same species from Nara in the Honshu Island in a monophyletic group, but obviously discriminated from other species of *Amblyomma* ticks as well other ixodid genera (*Haemaphysalis*, *Archaeochroton*, *Bothriocroton*, *Dermacentor*, *Robertsicus*, and *Rhipicephalus*) of ticks.

The results of polymorphic sites comparison showed that relying solely on one single mitochondrial gene for phylogenetic analysis of *A. testudinarium* might yield incorrect or incomplete conclusions (Table 3). Currently, most studies focusing on population structure and phylogenetic analysis of ticks build the final conclusions based on the results obtained by targeting more than one mitochondrial genes (Gui et al., 2021; Regilme et al., 2021). Furthermore, we showed that *A. testudinarium* population in Japan is genetically more diverse than the closely related *Amblyomma* sp. of Myanmar. In fact, the evolution of mitochondrial genes can be affected by several ecological factors such as endosymbionts and animal host diversity (Kaufman et al., 2018; Sassera et al., 2006). The difference in diversity between *A. testudinarium* in Japan and *Amblyomma* sp. in Myanmar could be attributed to the greater breadth of sampling collection sites in Japan compared to Myanmar where we collected the samples from one site. Unfortunately, we did not investigate the bacterial endosymbionts in our study, so we suggest that more studies are required to correlate the endosymbiont genetics and the mitogenome divergence in *A. testudinarium*.

*Amblyomma testudinarium* from China (accession number: MT029329) showed low sequence similarity with those from Japan but relatively high similarity with *A. javanense* reported from China (Table 2). This was supported by the analysis based on the 16S rDNA obtained from *A. testudinarium*, which included samples from Taiwan, Japan, and Thailand reported by other studies (Chao et al., 2017; Malaisri et al., 2015). We confirmed that the sequence from China (MT029329) is different from *A. testudinarium* from Japan, Taiwan, and Thailand (Figure 2). Collectively, these data suggest a possibility of misidentification of the tick specimen used in the previous report (Chang et al., 2020) or the presence of cryptic species related to *A. javanense* in China.

The 16S rDNA sequences of *A. testudinarium* from Japan are clustered together with those from Taiwan but not with Thailand (Figure 2). These findings suggest that natural aquatic barriers around Japan lowered the chance of *A. testudinarium* gene flow between East and Southeast Asia. This scenario was previously suggested for *I. ricinus* in continental Europe, where marine barriers around the British Isles were suggested to prevent the gene flow of *I. ricinus* (Al‐Khafaji et al., 2019). Our results also showed that a possible gene flow of *A. testudinarium* between Japan and Taiwan might have occurred presumably through the migratory birds flying through the East‐Asia Australian flyway (EAAF).

In general, geographical variations are mostly accompanied with differences in the distribution of tick hosts. The large animals are the primary hosts for *A. testudinarium*, while larvae and nymphs feed on small mammals, birds, reptiles, and amphibians (Kitaoka, 1974; Takada, 1990; Yamaguti et al., 1971). In Japan, adult *A. testudinarium* are frequently found on wild boars, which habitats are linked to those of *A. testudinarium* (Motoi et al., 2013). The rapid expansion of the wild boar habitats (Kodera et al., 2001) may be the reason for no clear geographic substructuring among *A. testudinarium* populations in Japan (Figure 6). However, future studies with larger numbers of *Amblyomma* samples collected from Japan and other countries are required to confirm the possible genetic distinction and pattern of geographical distribution of *A. testurinarium* from Amami island, Miyazaki, and Ehime prefectures.

The genetic variation within *A. testudinarium* populations in Japan, that was detected by the AMOVA statistics, could be attributed to selective pressures due to several ecoclimatic and environmental changes that requires adaptive genetic modifications. According to the Japan Meteorological Agency (https://www.data.jma.go.jp/gmd/cpd/longfcst/en/tourist.html), the climate in Japan ranges from subarctic in the north to subtropical in the south with Okinawa and Amami having humid and hot summers and mild winters compared to the warm summers and very cold winters in the remaining regions. It was reported previously that dynamics of arthropod vectors including ticks and the associated vector‐borne diseases are sensitive to climate change (Githeko et al., 2000). We also detected a pattern for the population differentiation of *A. testudinarium* in accordance with the spatial isolation where specimens collected from Amami island might have its own population in Japan. This finding could be explained by the complete geographical separation from the Japan mainland. The climate around Amami island is humid subtropical and known as Köppen climate classification (Cfa) with high precipitation throughout the year. Furthermore, the life cycle of *A. testudinarium* in Amami island is not clear; however, it is possible that this tick species utilizes endogenous animal species of this island as feeding hosts (Chae et al., 2017). Any of these factors may have supported the distinction of *A. testudinarium* tick population in Amami island.

The algorithms in our study support the expansion history of *A. testudinarium* in Japan. We detected a relatively high haplotype diversity in Japan, suggesting a strong possibility of sudden expansion (Amzati et al., 2018; Ray et al., 2003; Simonsen et al., 1995). By contrast, *Amblyomma* sp. in Myanmar was suggested to have a bottleneck historical event. Although our study did not estimate this sudden expansion and bottleneck times, relatively high diversity of *A. testudinarium* in Japan within population indicates that *A. testudinarium* colonies have been existed in Japan for long time. However, our study included samples that were collected from only a single sampling site in Myanmar, and we strongly suggest further investigations using *A. testudinarium* and the closely related *Amblyomma* spp. from all over the country to find a possible explanation of the population equilibrium observed in our study. Our finding that *A. testudinarium* in Japan has experienced a sudden expansion event agrees with the expected movement of migratory birds that are considered the carrying hosts of ticks in the country (Ishiguro et al., 2000; Kuo et al., 2017). This can be supported by the possible establishment of new colonies in previously unoccupied geographical locations in Japan, such as the recent detection of *A. testudinarium* in Hokkaido Island for the first time in history (Nakao et al., 2021).

## 5 CONCLUSION

Overall, we report whole mitogenome sequences of 30 *A. testudinarium* from Japan and 9 closely related *Amblyomma* species from Myanmar. Our study provides the first molecular evidence and convincing sequences to confirm the genetic identity and diversity of *Amblyomma* sp. in Myanmar. The lack of geographic substructuring among *A. testudinarium* populations in Japan suggests that geographic distance might not have an impact on the genetic variation. The data provides information about the sudden expansion event of *A. testudinarium* in Japan. With the advancement of high‐throughput sequencing techniques and bioinformatics, phylogeographic approaches can be used to distinguish *A. testudinarium* globally based on the genetic data of the whole mitochondrial genomes. The accumulation of more mitogenome sequences from different geographic regions can help in understanding the phylogeny of *Amblyomma* ticks present in East and Southeast Asia.

## CONFLICT OF INTEREST

The authors have no competing interests to declare.

## Supporting information


Table S1

## Data Availability

The data that support the findings of this study are openly available in the DNA Data Bank of Japan (http://www.ddbj.nig.ac.jp) with accession numbers of LC554761‐LC554790 (samples from Japan) and LC633546‐LC633554 (samples from Myanmar).
